# Association between Attitude towards Wife Beating and Childhood Diarrhea: A Demographic and Health Survey-Based Study in 25 Sub-Saharan African Countries

**DOI:** 10.1155/2021/4870994

**Published:** 2021-11-13

**Authors:** Betregiorgis Zegeye, Mpho Keetile, Bright Opoku Ahinkorah, Edward Kwabena Ameyaw, Abdul-Aziz Seidu, Sanni Yaya

**Affiliations:** ^1^Shewarobit Field Office, HaSET Maternal and Child Health Research Program, Addis Ababa, Ethiopia; ^2^Department of Population Studies, University of Botswana, Private Bag 00705, Gaborone, Botswana; ^3^The Australian Centre for Public and Population Health Research (ACPPHR), Faculty of Health, University of Technology Sydney, Ultimo, Australia; ^4^Department of Population and Health, University of Cape Coast, Cape Coast, Ghana; ^5^University of Parakou, Faculty of Medicine, Parakou, Benin

## Abstract

**Background:**

Childhood diarrhea remains a major public health problem in sub-Saharan Africa (SSA). Women empowerment reduces child mortality, and wife beating attitude is one of the indicators of women empowerment. There is a dearth of evidence about wife beating attitudes and childhood diarrhea in SSA. Therefore, the present study aimed to examine the association between attitude towards wife beating and diarrhea among under-five children.

**Methods:**

We used Demographic and Health Surveys from 25 countries in SSA that were conducted between 2010 and 2020. Using Stata version 14 software, we carried out the analysis on 153,864 children under five. Bivariate and multivariate logistic regression analyses were applied, and the results were presented using adjusted odd ratios (aOR) at 95% confidence interval (CI).

**Results:**

The pooled results show that 71.4% of married women disagreed with wife beating. About 20.5% of under-five children of married women had diarrhea. Childhood diarrhea varied from highest prevalence in Chad (27.9%) to the lowest prevalence in Sierra-Leone (8.5%). The study showed lower odds of diarrhea among children of married women who disagreed with wife beating (aOR = 0.66 95% CI; 0.54–0.80) compared to children of married women who agreed with wife beating. Moreover, the study results show that women's age (35–39 years-aOR = 0.48, 95% CI; 0.31–0.74, 40–44 years-aOR = 0.57, 95% CI; 0.35–0.93, 45–49 years-aOR = 0.35, 95% CI; 0.16–0.79) was negatively associated with childhood diarrhea, while husband's education (primary school-aOR = 1.36, 95% CI; 1.05–1.77), parity (ever born 3-4 children-aOR = 1.36, 95% CI; 1.09–1.70, and 5+ children-aOR = 1.56, 95% CI; 1.14–2.12), and religion (Muslim-aOR = 3.56, 95% CI; 1.44–8.83) were positively associated with diarrhea among under-five children.

**Conclusions:**

The study shows association between women attitude towards wife beating and childhood diarrhea. Therefore, empowering women, especially young women by increasing awareness about domestic violence, their rights, and empowering them through education and economic advancement need to be considered in order to reduce childhood diarrhea. Moreover, fertility control or birth spacing and working closely with religious leaders are important factors to consider in reducing childhood diarrhea.

## 1. Introduction

Globally, substantial progress has been made in reducing death among children under five [1, 2], from 12.6 million in 1990 to 5.2 million in 2019 [1]. As of 2019, approximately 5.2 million under-five children died from preventable and treatable causes globally [1]. Sub-Saharan Africa (SSA) and Central and Southern Asia accounted for more than 80% of the 5.2 million under-five deaths in 2019, and jointly they account for 52% of the global under-five child mortality. SSA remains the region with the highest under-five mortality [2], with 1 child in 13 dying before his or her fifth birthday [1]. Diarrhea is the leading killer of children, and it accounted for approximately 8% of all deaths of under-five children in 2017.

Women empowerment is one of the mechanisms that can be used to improve child's health and reduce under-five mortality [3, 4]; in addition, it has positive influence on personal, familial, societal, and country's development [3, 4]. Different scholars have used several measurements for women empowerment such as economic, sociocultural, legal, political, and psychological measures [5–7].

In Demographic and Health Survey (DHS), attitude towards wife beating is one of the indicators of women empowerment [8, 9]. Few previous studies have reported an association between attitude towards wife beating and child health [7, 10–13]. For instance, studies in Benin [7] and SSA have shown that there is a positive influence of women empowerment on child feeding practices and nutritional status [11]. Another study in SSA by Yaya et al. showed that empowering women can reduce childhood malnutrition [12]. Women empowerment's influence on the uptake of child health services is also documented in Zimbabwe [14] and other low- and middle-income countries [13].

Evidence in SSA reported that child's characteristics such as child's age, size, and sex, maternal and/or household socioeconomic status, hygiene, and sanitation condition, and geographic related factors were associated with childhood diarrhea [15–18]. Meanwhile, one study in Indonesia has shown an association between attitude towards wife beating and childhood diarrhea [19]. However, the association between attitude towards wife beating and childhood diarrhea is not well studied in SSA. Therefore, the present study aimed to examine the association between attitude towards wife beating and diarrhea among under-five children in 25 countries in SSA.

## 2. Methods

### 2.1. Data Source

We extracted DHS data from 25 countries in SSA for analysis in this study (Table 1). DHS are nationally representative surveys aimed to collect data for monitoring demographic and several health indicators including women empowerment and childhood diarrhea [20]. These surveys are carried out in low- and middle-income countries with the financial and technical support of United States Aids for International Development (USAID) and International Child Fund (ICF), respectively [21].

DHS applied a stratified two-stage cluster sampling technique. In the first stage, Enumeration Areas (EA) were selected systematically from the sampling frame prepared from the recent national population census. Then, in the second stage, fixed numbers of households (usually 25–30 households) were selected using Probability Proportional to Size (PPS) technique [22]. Based on our inclusion criteria (country with DHS between 2010 and 2019, availability of dataset that had the variables of interest), we included 25 countries. We used the Individual Recode (IR) file for the analysis and the data are freely available at https://dhsprogram.com/data/available-datasets.cfm. We followed the guidelines of Strengthening of Observational Studies in Epidemiology (STROBE) during preparation of this manuscript [23].

**Figure 1:**
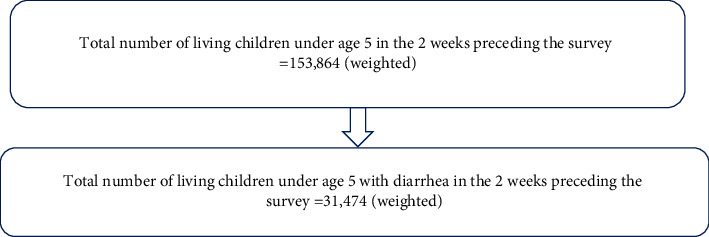
Schematic representation of sampling procedures: evidence from 25 SSA countries DHSs.

As shown in Figure 1, a total of 213,151 living children under age 5 were included in the surveys. Of them, 153,864 had diarrhea in the 2 weeks preceding the survey, and all are included for analysis of this study (Figure 1).

### 2.2. Study Variables

#### 2.2.1. Outcome Variable

The outcome variable of the study was childhood diarrhea. The number of living children under age of 5 with diarrhea in the 2 weeks preceding the survey was included [24, 25]. According to WHO, diarrhea is defined as the passage of three or more loose or liquid stools per day [26]. For this study, we included only under-five children of married women.

#### 2.2.2. Independent Variable

The independent variable of interest for this study was attitude towards wife beating. In DHS, married women aged 15–49 who disagree/not justify for all of five wife beating reasons, burning food, arguing with husband, going out without telling husband, neglecting the children, and refusing to have sexual intercourse with husband, are considered as empowered. However, if the married women agree/justify for at least one of above-mentioned wife beating reasons, they are not considered as empowered. Based on this concept, we categorized/coded them as 1 if the married women disagreed/not justified wife beating for all five reasons, and we coded them as 0 if the women agreed/justified for at least one of the five above-mentioned wife beating reasons [8, 27–30].

#### 2.2.3. Control Variables

Based on previous studies [15–18], we considered eleven control variables that influence the outcome variable such as age in years [11, 15–48], women's educational level (no formal education, primary school, secondary school, and higher), husband educational level (no formal education, primary school, secondary school, and higher), women's occupation (not working, professional/technical/managerial, agricultural, manual, and others), wealth index (poorest, poorer, middle, richer, and richest), media exposure (no and yes), place of residence (urban and rural), parity (≤2, 3-4, and 5+), decision making (no and yes), barriers to healthcare access (no and yes), and religion (Christian, Muslim, and others).

### 2.3. Statistical Analyses

Using Stata version 14 software, the analysis was carried out using the following steps. First, descriptive analyses, such as frequencies of the dependent variable, independent variable, and control variables, were done and presented using table and graphs. Then, bivariate logistic regression analyses were conducted to select candidate control variables at *p* value less than or equal to 0.15 (*p* ≤ 0.15). We considered this to include many confounders [31, 32]. Then, multicollinearity test was conducted using Variance Inflation Factor (VIF) to check whether there was collinearity among the independent and/or control variables and we discovered that there was no evidence of collinearity (mean VIF = 1.90, Min VIF = 1.00, Max VIF = 3.77). Finally, multivariate logistic regression analysis was conducted by including all control variables that were selected at the bivariate analysis, along with the independent variable to check the association between wife beating attitude (disagreed/not justified for wife beating) and childhood diarrhea. The model fitness was checked using Hosmer–Lemeshow and confirmed that the model was fit (*p* = 0.8735). The results were presented using crude odd ratio (COR) and adjusted odd ratio (AOR), at 95% confidence interval (CI). To take care of the complex nature DHS's data, we used the “svyset” command during analysis, and all three design elements, that is, weight, cluster, and strata, were taken into consideration.

### 2.4. Ethical Clearance

We used publicly available secondary data for analysis of this study (available at: https://dhsprogram.com/data/available-datasets.cfm). Since ethical procedures are the responsibility of institutions that funded, commissioned, and managed the surveys, further ethical clearance was not required. ICF international approved that all the DHS surveys follow the US Department of Health and Human Services rules, for respecting of human subject's rights. For more details related to ethical issues, readers can visit http://goo.gl/ny8T6X.

## 3. Results

### 3.1. Sociodemographic Characteristics

In total, 153,864 under-five children of married women/care givers were involved in this analysis. Regarding respondents, from this total, about 7.9% were within the age groups of 15–19 years, while 35.3% of the respondents were rural residents. More than one-quarter (27.5%) of the respondents and one-fifth (21.1%) of their husbands had no formal education. Regarding wife beating attitude, approximately 34.6% married women did not decide by themselves or together with their husband on at least one of the three decision-making parameters: their own health, to purchase large household expenses, and to visit families/relatives. 28.6% of married women justified wife beating practice by their husband for at least one of the five wife beating reasons: going out without telling their husband, arguing with their husband, neglecting children, burning foods, and refusing to have sex with their husband. However, 71.4% of married women did not justify/accept wife beating in any of the five wife beating reasons.

### 3.2. Prevalence of Disagreement with Wife Beating

**Figure 2:**
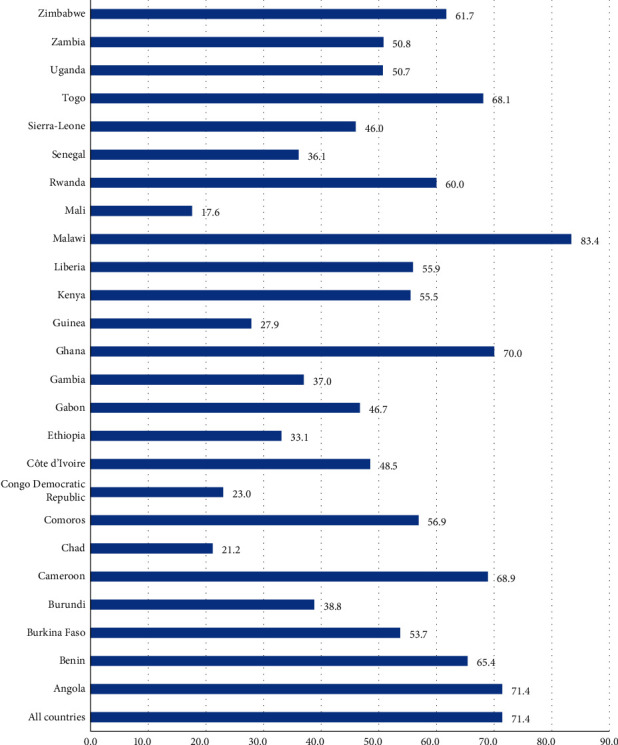
Prevalence of disagreement with wife beating among married women of children under five in SSA.

The pooled result shows that about 71.4% (95% CI; 69.3%–73.4%) of married women in the 25 countries in SSA disagreed with all of the five wife beating reasons, with the highest (83.4%) and lowest (17.7%) prevalence in Malawi and Mali, respectively (Figure 2).

### 3.3. Distribution of Prevalence of Childhood Diarrhea across Explanatory/Control Variables

As shown in Table 2, prevalence of childhood diarrhea varied across subpopulations of explanatory variables. For instance, prevalence of childhood diarrhea among children from married women who were empowered was 18.3%, while it was 25.6% among married women who were not empowered. Similarly, childhood diarrhea varied across married women's age. For instance, about 25% of under-five children from married women within the age groups of 20–24 years had diarrhea, while about 11.9% of under-five years children had diarrhea among married women who were within 44–49 years of age. Prevalence of childhood diarrhea also varied based on religious beliefs. For instance, approximately 45.7% of under-five children had diarrhea, but the prevalence was lowered to about 18.3% and 20.5% among under-five children who belonged to other religions and Christianity, respectively (Table 2).

### 3.4. Prevalence of Childhood Diarrhea

**Figure 3:**
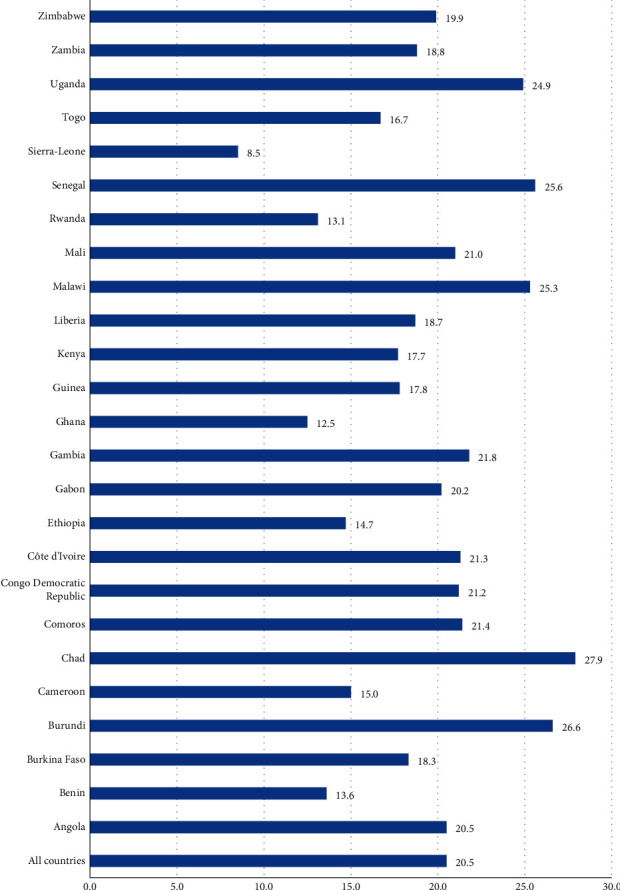
Prevalence of diarrhea among children under five of married women in sub-Saharan Africa.

As shown in Figure 3, the pooled results from the 25 sub-Saharan African countries show that about 20.5% (95% CI; 18.7%–22.3%) of under-five years of age children from married women had diarrhea. Prevalence of childhood diarrhea varied across studied countries. The study shows that the highest prevalence of childhood diarrhea was observed in Chad (27.9%), Burundi (26.6%), Liberia (26%), Senegal (25.6%), Malawi (25.3%), and Uganda (24.9%), respectively. On the other hand, lowest childhood diarrhea was observed in Sierra-Leone (8.5%), Ghana (12.5%), Rwanda (13.1%), Benin (13.6%), and Ethiopia (14.8%), respectively (Figure 3).

### 3.5. Association between Women's Attitude towards Wife Beating and Childhood Diarrhea

Table 3 shows the association between attitude towards wife beating and childhood diarrhea. More specifically, the study shows lower odds of diarrhea among under-five children of married women who disagreed with wife beating (aOR = 0.66 95% CI; 0.54–0.80) compared to under-five children of married women who agreed with wife beating. The study also shows lower odds of diarrhea among under-five children of married women within the age groups of 35–39 years (0.48, 95% CI; 0.31–0.74), 40–44 years (aOR = 0.57, 95% CI; 0.35–0.93), and 45–49 years (aOR = 0.35, 95% CI; 0.16–0.79) compared to under-five children of married women within the age groups of 15–19 years.

Moreover, the study shows higher odds of diarrhea among under-five children of married women whose husband had attended primary school (aOR = 1.36, 95% CI; 1.05–1.77) compared to under-five children of married women whose husbands did not attend formal education. In this study, we observed higher odds of diarrhea among under-five children of married women who ever gave birth to 3-4 children (aOR = 1.36, 95% CI; 1.09–1.70) and 5+ children (aOR = 1.56, 95% CI; 1.14–2.12) compared to under-five children of married women who ever gave birth to less than or equal to two children. Finally, we found higher odds of diarrhea among under-five children of married women who belonged to Islam (aOR = 3.56, 95% CI; 1.44–8.83) compared to under-five children of married women who belonged to the Christianity.

## 4. Discussion

To the best of our knowledge, this is the first study to examine the association between married women's attitude towards wife beating and childhood diarrhea in SSA. The pooled results from the 25 sub-Saharan African countries show that about 71.4% of married women were empowered (disagreed with wife beating), and about 20.5% of under-five children of married women had diarrhea. This finding is higher compared to previous studies in Ethiopia (14.9%) [33] India (9%) [34] and SSA (16%) [16]. Higher prevalence of childhood diarrhea among children of married women in the present study could be due to variations in the methodology we used including the target population [35, 36]. We only included married women because maternal and child health services are more challenging among married women [35, 36]. This might be for the reason that domestic violence and poor decision-making power [35, 36], financial dependency on men, and the need of husband's consent for access and utilization of healthcare services [35].

In this study, we found lower odds of diarrhea among under-five children of married women who disagreed with wife beating compared to under-five children of married women who agreed with wife beating. Comparable findings were reported in prior studies in Indonesia [19]. Lower odds of diarrhea among under-five children of married women who disagreed/not justified for wife beating could be due to higher decision-making power [37], better socioeconomic status [38, 39], health seeking behavior, and utilization of child health services, such as immunization [13, 40] and child feeding practices [7, 11, 12, 41]. Evidence shows that women who disagree/not justified wife beating usually utilize maternal health services, such as antenatal care, health facility delivery, and postnatal care [36, 42]. Better care of children and health seeking behavior for child health services are seen among women who usually utilize maternal health services and visit health institutions [43].

We found that the odds of diarrhea were lower among under-five children of older married women compared to under-five children of adolescent married women as documented in SSA [16] and Pernambuco, Brazil [44]. This could be because younger women might be inexperienced in care of children including feeding of their children compared to older women [16, 44, 45]. Again, this experience might help the women to have more knowledge and demonstrate their knowledge in prevention of diarrhea [16].

In contrast with a previous study in Ethiopia [46], the present study shows that there were higher odds of diarrhea among under-five children of married women whose husband attended primary school compared to under-five children of married women whose husband had no formal education. This may need further studies to know the mechanism why higher odds of childhood diarrhea are seen among women with educated husband.

This study shows that the odds of diarrhea were higher among under-five children of married women with higher paritycompared to under-five children of married women with lower parity . This could be due to the effect of high parity itself and their relations with short birth interval deplete maternal nutrition and child survival for infectious diseases [47]. The physical and caloric demands, mixed with physical and caloric stress life in repeated pregnancy result in depletion of maternal nutritional status [48]. The other justification could be children from high parity women usually become nutritionally poor and vulnerable for infection because of smaller parental food security and/or competition among family members or siblings for limited resources [48].

Moreover, the present study showed the association between religious beliefs and childhood diarrhea [34, 49, 50]. This finding is comparable with previous studies in India [34, 49], Kenya, Nigeria, and Niger [50] that showed the association between religion and healthcare seeking behavior for childhood diarrhea. This could be linked with the women's religious beliefs/perceptions as well as religious authorities' prohibition to modern treatment seeking from health facilities [49, 50]. More specifically, we found higher odds of diarrhea among under-five children of married women who belonged to Islamic religion compared to under-five children of married women who professed to be Christian. A previous study in India showed that the odds of childhood diarrhea were 18% higher among children who belonged to Islamic religion compared to Hindu [34]. Previous study in SSA [51] showed that child mortality was also significantly higher among Muslim women compared to non-Muslim women. Meanwhile, in Nigeria, in the study on women with at least one live birth, 43% of Muslims had at least one child death and 29% among non-Muslims [51]. This could be related to utilization of maternal and child health services [51] and lower years of schooling among Muslim women compared to non-Muslim women [51]. A prior study in SSA showed the large difference in years of schooling from 9.2 years to 3.2 years between non-Muslim and Muslim women, respectively [51]. Not only among Muslim women has evidence showed that generally the majority of Muslim adults in SSA lack basic educational attainment [52, 53], but also different prior studies in India [34], Bangladesh [54], and different parts of Ethiopia [55, 56] documented that better childcare, feeding, and hygiene practice are associated with women education.

The other possible reason for higher odds of childhood diarrhea among children of Muslim women could be linkage between nonacceptance and poor utilization of vaccination, such as Rota virus [57]. However, WHO delivered a report on the joint view of Islamic religious academics, saying that animal-derived medical products, including vaccines, that undergo a transformation are considered clean [58]. Not all of the Islamic faith considers vaccines to be halal or allowable under Islamic Shariah Law [57]. In situations where vaccine is considered haram (banned), children might not get immunization services and still vulnerable for life threatening disease and need working with religious leaders especially where vaccines are strictly prohibited [57].

### 4.1. Strengths and Limitations of the Study

Using nationally representative and large sample size, examining attitude towards wife beating and childhood diarrhea in 25 countries in SSA is a major strength of the paper. However, the study has the following limitations. First, the cross-sectional nature of DHS data might not allow inferring cause-effect relationships. Second, since the data were self-reported, recall bias might affect the findings. Third, since we included only married women, the findings might not be applicable to all reproductive age [11, 15–48] women and finally, because of exclusion criteria few countries in SSA were not included and the findings might not be applicable to all sub-Saharan African countries.

## 5. Conclusion

The findings from the pooled results show that a large proportion of married women (71.4%) disagreed with all the five reasons for wife beating and one-fifth of under-five children had diarrhea. Childhood diarrhea varied from a highest prevalence in Chad (27.9%) to a lowest prevalence in Sierra-Leone (8.5%). We found lower odds of childhood diarrhea among married women who disagreed with wife beating compared to children of married women who agreed with wife beating. Moreover, the study shows that women's age, husband's education, parity, and religion were associated with diarrhea among under-five children.

Therefore, in order to improve prevalence ofdiarrhea among children under five in SSA, the national government in each country and other stakeholders who are concerned for child health need to work on women empowerment. The interventions may be implemented through multidimensional mechanisms including education, and economy and other strategies, such as increasing awareness about domestic violence and their reproductive and human rights, especially for young women. Furthermore, strengthening of family planning services or birth spacing and working closely with religious leaders also are important to reduce childhood diarrhea.

## Figures and Tables

**Table 1:** Year of survey and weighted sample in each studied country.

Country	Year of survey	Weighted sample
Angola	2015/16	6,167
Benin	2017/18	7,979
Burkina Faso	2010	9,450
Burundi	2016/17	7,385
Cameroon	2018/19	4,798
Chad	2014/15	9,726
Comoros	2012	1,825
Congo Democratic Republic	2013/14	9,107
Cote devoir	2011/12	4,244
Ethiopia	2016	6,417
Gabon	2012	2,767
Gambia	2013	4,860
Ghana	2014	3,542
Guinea	2018	4,823
Kenya	2014	12,007
Liberia	2019/20	2,815
Malawi	2015/16	10,758
Mali	2018	5,809
Rwanda	2014/15	4,640
Senegal	2010/11	7,255
Sierra-Leone	2019	5,724
Togo	2013/14	4,387
Uganda	2016	8,123
Zambia	2018/19	5,335
Zimbabwe	2015	3,921
Total		153,864

**Table 2:** Frequency distribution of respondents and distribution of childhood diarrhea cross explanatory variables: evidence from 25 SSA countries.

Variables	Frequency (weighted %)	Childhood diarrhea (weighted %)	
		No	Yes
Are women empowered			
No	77,421 (29.41)	74.39	25.61
Yes	71,149 (70.59)	81.69	18.31
Age in years			
15–19	9,115 (7.86)	79.96	20.04
20–24	33,137 (19.88)	75.04	24.96
25–29	41,127 (21.60)	78.3	21.70
30–34	32,813 (16.88)	80.78	19.22
35–39	23,473 (14.56)	84.59	15.41
40–44	11,579 (11.73)	82.28	17.72
45–49	3,661 (7.51)	88.12	11.88
Women's educational level			
No formal education	67,559 (27.57)	79.72	20.28
Primary school	52,631 (39.40)	78.45	21.55
Secondary school	30,359 (29.58)	79.94	20.06
Higher	4,349 (3.45)	87.23	12.77
Husband educational level			
No formal education	59,036 (21.44)	83.10	16.90
Primary school	40,825 (27.08)	76.98	23.02
Secondary school	39,195 (44.61)	79.34	20.66
Higher	8,888 (6.87)	79.90	20.10
Women's occupation			
Not working	41,443 (25.79)	81.75	18.25
Professional/technical/managerial	4,611 (4.64)	81.52	18.48
Agricultural	56,360 (30.40)	79.28	20.72
Manual	10,342 (3.16)	77.10	22.90
Others	35,783 (36.02)	78.15	21.85
Wealth index			
Poorest	38,027 (18.36)	80.66	19.34
Poorer	33,570 (21.96)	78.06	21.94
Middle	30,749 (21.52)	78.98	21.02
Richer	27,703 (20.03)	79.34	20.66
Richest	24,856 (18.14)	81.11	18.89
Place of residence			
Urban	46,125 (63.85)	79.68	20.32
Rural	108,780 (36.15)	79.30	20.70
Parity			
≤2	54,446 (31.71)	77.43	22.57
3-4	47,247 (31.54)	80.72	19.28
5+	53,212 (36.75)	80.28	19.72
Decision making			
No	89,153 (35.44)	77.35	22.65
Yes	59,375 (64.56)	80.75	19.25
Barriers to healthcare access			
No	46,486 (30.33)	80.07	19.93
Yes	95,713 (69.67)	79.32	20.68
Religion			
Christian	90,493 (93.22)	79.47	20.53
Muslim	56,720 (0.31)	54.35	45.65
Others	7,565 (6.48)	81.74	18.26
Media exposure			
No	73,283 (29.25)	81.51	18.49
Yes	81,272 (70.75)	78.73	21.27

**Table 3:** Association between wife beating attitude and childhood diarrhea among married women: evidence from 25 SSA countries.

Variables	cOR (95% CI)	*p* value	aOR (95% CI)	*p* value
Women are empowered				
No	Ref		Ref	
Yes	0.65 (0.53–0.78)	**<0.001**	0.66 (0.54–0.80)	**0.001**
Control variables				
Age in years				
15–19	Ref		Ref	
20–24	1.32 (0.97–1.80)	**0.074**	1.21 (0.89–1.64)	0.218
25–29	1.10 (0.80–1.51)	0.530	0.86 (0.61–1.23)	0.434
30–34	0.94 (0.64–1.40)	0.793	0.68 (0.44–1.04)	0.076
35–39	0.72 (0.49–1.07)	0.111	0.48 (0.31–0.74)	**0.001**
40–44	0.85 (0.55–1.33)	0.498	0.57 (0.35–0.93)	**0.024**
45–49	0.53 (0.25–1.12)	0.097	0.35 (0.16–0.79)	**0.012**
Women's educational level				
No formal education	Ref			
Primary school	1.07 (0.85–1.35)	0.511	NA	NA
Secondary school	0.98 (0.78–1.23)	0.902	NA	NA
Higher	0.57 (0.21–1.57)	0.282	NA	NA
Husband educational level				
No formal education	Ref		Ref	
Primary school	1.47 (1.13–1.89)	**0.003**	1.36 (1.05–1.77)	**0.020**
Secondary school	1.28 (1.03–1.57)	**0.020**	1.19 (0.95–1.49)	0.118
Higher	1.23 (0.76–1.99)	0.385	1.33 (0.80–2.22)	0.257
Women's occupation				
Not working	Ref		Ref	
Professional/technical/managerial	1.01 (0.60–1.70)	0.955	1.28 (0.77–2.13)	0.324
Agricultural	1.17 (0.92–1.47)	0.182	1.22 (0.94–1.60)	0.129
Manual	1.33 (0.80–2.21)	0.270	1.35 (0.81–2.25)	0.237
Others	1.25 (0.96–1.63)	**0.096**	1.30 (1.00–1.70)	**0.046**
Wealth index				
Poorest	Ref			
Poorer	1.17 (0.91–1.49)	0.201	NA	NA
Middle	1.10 (0.86–1.43)	0.419	NA	NA
Richer	1.08 (0.78–1.49)	0.613	NA	NA
Richest	0.97 (0.69–1.34)	0.861	NA	NA
Media exposure				
No	Ref		Ref	
Yes	1.19 (0.98–1.43)	**0.065**	1.25 (0.99–1.57)	**0.057**
Place of residence				
Urban	Ref			
Rural	1.02 (0.83–1.25)	0.821	NA	NA
Parity				
≤2	Ref		Ref	
3-4	1.18 (0.98–1.43)	**0.075**	1.36 (1.09–1.70)	**0.006**
5+	0.97 (0.75–1.25)	0.829	1.56 (1.14–2.12)	**0.005**
Decision making				
No	Ref		Ref	
Yes	0.81 (0.66–1.00)	**0.052**	0.83 (0.68–1.01)	0.077
Barriers to healthcare access				
No	Ref			
Yes	1.04 (0.84–1.29)	0.663	NA	NA
Religion				
Christian	Ref		Ref	
Muslim	3.25 (1.37–7.67)	**0.007**	3.56 (1.44–8.83)	**0.006**
Others	0.86 (0.58–1.26)	0.458	0.89 (0.60–1.32)	0.578

cOR: crude odd ratio, aOR: adjusted odd ratio, Ref: reference, and NA: not included variable due to being not significant at bivariate logistic regression. Bold values denote statistical significance at the p < 0.05 level.

## Data Availability

Data for this study were sourced from Demographic and Health Surveys (DHS) and are available here http://dhsprogram.com/data/available-datasets.cfm.
